# Differences in adjustment by child developmental stage among caregivers of children with disorders of sex development

**DOI:** 10.1186/1687-9856-2011-16

**Published:** 2011-11-10

**Authors:** Stephanie E Hullmann, David A Fedele, Cortney Wolfe-Christensen, Larry L Mullins, Amy B Wisniewski

**Affiliations:** 1 Department of Psychology, Oklahoma State University, Stillwater, Oklahoma, 74078, USA; 2 Department of Pediatric Urology, Children's Hospital of Michigan, Detroit, Michigan, 48201, USA; 3 Department of Urology, University of Oklahoma Health Sciences Center, Oklahoma City, Oklahoma, 73104, USA

**Keywords:** disorder of sex development, parenting stress, overprotection

## Abstract

**Background:**

The current study sought to compare levels of overprotection and parenting stress reported by caregivers of children with disorders of sex development at four different developmental stages.

**Methods:**

Caregivers (*N *= 59) of children with disorders of sex development were recruited from specialty clinics and were asked to complete the Parent Protection Scale and Parenting Stress Index/Short Form as measures of overprotective behaviors and parenting stress, respectively.

**Results:**

Analyses of covariance (ANCOVAs) were conducted to examine differences between caregiver report of overprotection and parenting stress. Results revealed that caregivers of infants and toddlers exhibited more overprotective behaviors than caregivers of children in the other age groups. Further, caregivers of adolescents experienced significantly more parenting stress than caregivers of school-age children, and this effect was driven by personal distress and problematic parent-child interactions, rather than having a difficult child.

**Conclusions:**

These results suggest that caregivers of children with disorders of sex development may have different psychosocial needs based upon their child's developmental stage and based upon the disorder-related challenges that are most salient at that developmental stage.

## Introduction

Disorders of sex development, or DSD, are a group of congenital medical conditions in which affected individuals experience discordance between their genetic, gonadal, and/or phenotypic sex [1]. Caring for a child with a chronic medical condition can be a significant stressor for parents such that they may experience guilt and uncertainty regarding their child's disease, treatment, and long-term prognosis [2]. Parents of children with DSD must make many decisions regarding the social, emotional, medical, and surgical management of their child's illness throughout the child's life, and these choices often vary depending on the age and developmental level of their child. For example, decisions about sex of rearing are typically made by caregivers of infants and young toddlers [1], whereas judgments about whether or not to pursue gonadectomy or genital surgery for their child may persist into adolescence [1,3,4]. Throughout the child's life, caregivers must also make decisions regarding how much and with whom to share information regarding the child's diagnosis (e.g., family members, childcare workers, teachers, and their child). Furthermore, as children mature into puberty, caregivers may be faced with choices regarding hormone therapy to support development of their child's secondary sexual characteristics.

Child development is often conceptualized as a multifaceted process that occurs in four relatively distinct stages based largely upon physical and mental development and degree of independence from caregivers. These stages include infancy and toddlerhood, preschool age, school age, and adolescence [5]. During the first three years of life (i.e., infancy and toddlerhood), children experience rapid physical development, and it is during this time that gender identity is established [6,7]. Though children continue to grow during the preschool- and school-age stages, their rate of growth slows. During adolescence, individuals, once again, undergo drastic physical changes when the hypothalamic-pituitary-gonadal (HPG) axis is reactivated, and they experience puberty [5]. Adolescence is also a time when children ostensibly learn to exert their independence from their family and further develop their autonomy [5] by developing close peer relationships and beginning to enter into romantic and sexual relationships. The major role of the HPG axis during pre- and peri-natal development in boys, and in adolescence for both boys and girls, combined with the development of gender and sexuality at these ages, may make the challenge of caring for a child with a DSD particularly salient to parents at these developmental stages.

Parental overprotection and parenting stress are both *parenting capacity variables *[8] that involve discrete aspects of a caregiver's psychosocial adjustment. Both of these constructs have been conceptualized as measures of adjustment within the parent-child relationship [9,10]. Parental overprotection describes overt behaviors on the part of the parent to protect their child. Importantly, these behaviors are considered excessive for the child's current developmental level [11]. On the other hand, parenting stress is a measure of the parent's stress regarding their role as a parent and their relationship with their child [12]. Both of these caregiver adjustment variables are related to adjustment outcomes of children with chronic illnesses. Specifically, parental overprotection has been shown to be related to poorer quality of life and higher rates of behavior problems in children with chronic illnesses [13-15]. Similarly, higher levels of parenting stress are related to poorer emotional, behavioral, and social adjustment and worse physical health in children with chronic illnesses [16-18]. Notably, age differences have been observed in both rates of overprotective behaviors and parenting stress such that caregivers of younger children are more overprotective and experience more parenting stress than caregivers of older children [11,12]. To date, few studies have examined these parenting capacity variables in caregivers of children with DSD. Notably, recent research with this population suggests that caregivers of children with DSD experience similar levels of these parenting capacity variables as caregivers of children with Type 1 diabetes mellitus [19]. Additionally, parenting stress and overprotection are related to medical factors such as genitoplasty and age at genitoplasty in children with DSD [20].

To date, no studies have examined the differential effect of the child's developmental stage on adjustment in caregivers of children with DSD. It stands to reason that, given the specific needs of children with DSD at each developmental stage, caregiver adjustment would change as the child's needs change and as the child's illness characteristics become more or less salient to the family. Specifically, we hypothesized that caregivers of infants and toddlers and caregivers of adolescents would exhibit the most overprotective behaviors and experience greater parenting stress than caregivers of children at other developmental stages.

## Methods

### Participants

Participants for the current examination included 59 caregivers (66.1% female) of children (69.5% girls) between the ages of .5 and 17.83 years (*M *= 5.54, *SD *= 4.75). Children in the current study had been diagnosed with 46,XX DSD due to 21-hydroxylase deficiency or transposition of the SRY gene reared female (*n *= 37) or 46,XY DSD due to androgen biosynthetic defects, androgen insensitivity or unknown causes reared male or female (*n *= 22). At birth, each child was assigned a Prader score describing the appearance of their external genitalia prior to medical and/or surgical intervention. Prader scores for females in the sample ranged from 1 to 5 (*M *= 2.78, *SD *= 1.10), and the scores ranged from 2 to 5 (*M *= 3.50, *SD *= .79) for males.

The caregivers in the sample were 19 to 47 years old (*M *= 34.24, *SD *= 7.16), and the majority of caregivers reported being married (63.8%). Additionally, 22.8% of the respondents reported an annual household income less than $20,000, 14% reported an income between $20,000 and $40,000, 24.6% reported an income between $40,000 and $60,000, and 38.6% reported an income over $60,000. With regard to race and ethnicity, the majority of participants self-identified as Caucasian (74.1%), 10.3% self-identified as African American, 5.2% as Native American, 5.2% as Hispanic, 3.4% as Asian American, and 1.7% as "other".

### Measures

The caregiver participants completed the following questionnaires as part of a larger study of psychosocial adjustment in caregivers of children with DSD. As such, part of this dataset was utilized in our earlier research [19,20].

#### Demographic Questionnaire

Parent participants completed a demographic questionnaire to provide information regarding their age, race, and marital status, their child's age, and their household income.

#### Parent Protection Scale

Parental overprotection was assessed using the Parent Protection Scale (PPS) [11]. The PPS, a 25-item, self-report measure, examines several dimensions of overprotective parenting behaviors. Caregivers are asked to rate the extent to which each statement is descriptive of their behavior with their child on a four-point scale ranging from *never *to *always*. A higher total score indicates a higher level of protective parenting behaviors. A score one standard deviation above the sample mean indicates clinically significant levels of overprotection [21]. Normative studies examining the PPS have demonstrated adequate internal reliability (.73) and moderate test-retest reliability (.86) [11]. The PPS has been utilized to measure protective parenting behaviors in a several different pediatric populations [17,22]. The internal reliability coefficient for the current sample was adequate (.71).

#### Parenting Stress Index/Short Form

The amount of stress present in the caregiver-child relationship was assessed using the Parenting Stress Index/Short Form (PSI/SF) [12]. The PSI/SF is a 36-item instrument that asks caregivers to rate the extent to which each statement is descriptive of their relationship with their child on a five-point scale ranging from *strongly agree *to *strongly disagree*. The PSI/SF yields a total parenting stress score as well as three subscale scores (i.e., stress attributable to the caregiver's personal distress, distress related to the child, and relational distress between the caregiver and child). Higher scores on all scales indicate higher levels of parenting stress. A total score of 90 or greater indicates clinically significant parenting stress. The PSI/SF is highly correlated with the full-length PSI instrument (*r *= .94), and the two-week test-retest reliability of the PSI with the PSI/SF is also very high (.95) [12]. The validity of the PSI and PSI/SF has been established in a range of populations, including parents of children with chronic illnesses [17,22-24]. The internal consistency for the current sample was excellent (.95).

### Procedure

Caregivers were invited to participate in the current study if they had a child who had been diagnosed with some type of 46,XX or 46,XY DSD. The study was IRB approved and all aspects of the projects were conducted in compliance with the APA ethical guidelines for research. For inclusion in the study, caregivers must have been at least 18 years old, able to provide informed consent, and able to read English at an 8^th ^grade reading level. All caregivers of these children were invited to participate; however, only data from one caregiver participant per child was used for the current study. For those families in which multiple caregivers participated (*n *= 33), one caregiver was randomly selected to be included in the analyses. Caregivers were recruited from their DSD specialty clinic at the University of Oklahoma Children's Hospital, Johns Hopkins Hospital, and the Women's and Children's Hospital of Buffalo. Additionally, caregivers of children with DSD receiving treatment at other hospitals who contacted the senior author regarding participation were included in the study.

All caregivers provided informed consent, and the study questionnaires were subsequently mailed to them for completion. Upon receipt of the study questionnaires, all caregivers were compensated with a $25 gift card to thank them for their participation. Three caregivers, one whose child has an XX, DSD and two whose children have an XY, DSD, declined participation, yielding a consent and completion rate of 97% for all caregivers approached to participate.

## Overview of Analyses

To determine whether levels of parental overprotection and parenting stress differed by a child's developmental level, an analysis of covariance (ANCOVA) was conducted, controlling for child and caregiver sex. Developmental level was divided into four categories based upon previously published guidelines [5]: 1) infants and toddlers (0 - 2 year-olds, *n *= 20), 2) preschool-age children (3 - 6 year-olds, *n *= 21), 3) school-age children (7 - 11 year-olds, *n *= 10), and 4) adolescents (12 - 18 year-olds, *n *= 8). Planned post-hoc comparisons using a Bonferroni alpha adjustment were conducted in order to examine pairwise differences among the marginal means while controlling for Type I error. The assumptions of homogeneity of regression slopes and variances were tenable for all analyses. Partial *η^2 ^*was used for an effect size estimate because it estimates the factor of interest while controlling for covariates.

## Results

### Preliminary Analyses

The sample was first examined to determine the percentage of caregivers meeting criteria for clinically significant levels of parenting stress and overprotective behaviors. In accordance with the authors' recommendations, a cutoff score of 90 on the PSI/SF [12] and 41 on the PPS (i.e., one standard deviation above the sample mean) [21] were utilized to identify clinically significant reports of parenting stress and overprotection, respectively. Please see Table 1 for clinical cutoff information by developmental level.

**Table 1:** Clinical Cut-off Percentages by Developmental Level

Variable	Overall *M*(*SD*)	Ages 0-2	Ages 3-6	Ages 7-11	Ages 12-18
Parental Overprotection	33.12(8.29)	58.80%	4.80%	0.00%	0.00%
Parenting Stress	72.68(21.69)	15.00%	23.80%	0.00%	37.5%

*Note*. Clinical cut-offs were ≥ 41 for Parental Overprotection and ≥ 90 for Parenting Stress.

### Caregiver Overprotection

**Figure 1:**
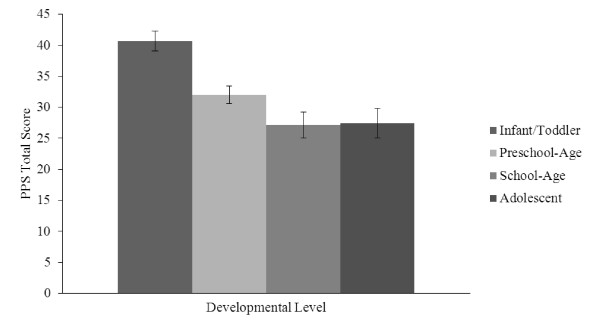
**Caregiver Overprotection by Developmental Level**.

Analyses demonstrated a significant main effect for developmental level, *F*(3,50) = 11.94, *p *<, 001, *η_p_*^2 ^= .42. Planned post-hoc comparisons revealed that, on average, caregivers of infants and toddlers (*M *= 40.64, *SEM *= 1.61, 95% CI [37.42, 43.87]) evidenced significantly higher levels of parental overprotection than caregivers of preschool-age children (*M *= 32.00, *SEM *= 1.44, 95% CI [29.11, 34.89], *p *= .001), school-age children (*M *= 27.14, *SEM *= 2.10, 95% CI [22.93, 31.35], *p *< .001), or adolescents (*M *= 27.46, *SEM *= 2.37, 95% CI [22.71, 32.21], *p *< .001). There were no other significant differences in parental overprotection between any of the other developmental levels (see Figure 1).

### Caregiver Stress

**Figure 2:**
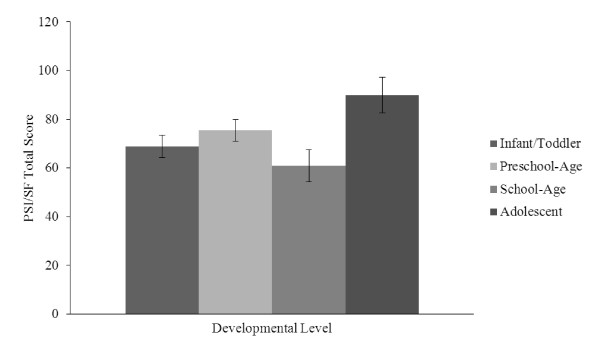
**Caregiver Stress by Developmental Level**.

Results revealed a significant main effect for developmental level, *F*(3,53) = 3.30, *p *= .027, *η_p_*^2 ^= .16. Planned post-hoc comparisons showed that, on average, caregivers of adolescents (*M *= 89.89, *SEM *= 7.34, 95% CI [75.17, 104.61]) reported significantly higher levels of stress than caregivers of school-age children (*M *= 60.89, *SEM *= 6.50, 95% CI [47.85, 73.93], *p *= .028]. There were no other significant differences in parenting stress between any of the other developmental levels (see Figure 2).

### Exploratory Analyses

Exploratory analyses sought to examine the subscales of the PSI/SF by the developmental levels. For the personal distress subscale, results revealed a significant main effect for developmental level, *F*(3,59) = 3.31, *p *= .036, *η_p_*^2 ^= .148. Planned post-hoc comparisons showed that, on average, caregivers of adolescents (*M *= 33.105, *SEM *= 2.714, 95% CI [27.662, 38.548]) reported significantly higher levels of personal distress than caregivers of infants and toddlers (*M *= 26.348, *SEM *= 1.696, 95% CI [22.947, 29.749], *p *= .041) and caregivers of school-age children (*M *= 22.207, *SEM *= 2.404, 95% CI [17.385, 27.028], *p *= .004). There were no other significant differences in caregiver personal distress between any of the other developmental levels. For the caregiver-child dysfunctional interaction subscale, results revealed a significant main effect for developmental level, *F*(3,59) = 3.291, *p *= .028, *η_p_*^2 ^= .157. Planned post-hoc comparisons revealed that, on average, caregivers of adolescents (*M *= 26.50, *SEM *= 2.63, 95% CI [21.23, 31.77]) reported significantly higher levels of caregiver-child dysfunctional interactions than caregivers of infants and toddlers (*M *= 18.40, *SEM *= 1.64, 95% CI [15.11, 21.69], *p *= .012), caregivers of preschool-age children (*M *= 19.13, *SEM *= 1.60, 95% CI [15.91, 22.34], *p *= .021), and caregivers of school-age children (*M *= 15.94, *SEM *= 2.33, 95% CI [11.27, 20.61], *p *= .004). There were no other significant differences in caregiver-child dysfunctional interaction between any of the other developmental levels. For the difficult child subscale, the main effect for developmental level was found to be nonsignficant, *p *> .05.

## Discussion

The current study sought to examine differences in levels of parental overprotection and parenting stress in caregivers of children with DSD across four developmental stages. Specifically, it was hypothesized that caregivers of infants and toddlers and caregivers of adolescents would exhibit the greatest rates of overprotective behaviors and parenting stress. The results partially supported this hypothesis in that caregivers of infants and toddlers were found to exhibit significantly more overprotective behaviors than caregivers of children in the other age groups. Caregivers of adolescents were not found to exhibit higher rates of overprotective behavior than the other groups. These results are consistent with the extant literature on parental overprotection, which states that caregivers of younger children exhibit more overprotective behavior than caregivers of older children [11]. In other words, developmental theory suggests that as children mature, they are expected to become more independent and autonomous [25]. As children grow more autonomous, caregivers may also have more opportunities to observe their child's resiliency, perhaps reducing the perceived benefits of their protective behavior. In this regard, caregivers of children with DSD do not differ from caregivers of children with other chronic illnesses in their ratings of parental overprotection.

Although speculative, it is possible that parental overprotection impacts decisions regarding their child's early medical care in a manner that is specific to DSD. For example, surgery to "normalize" ambiguous genitalia may be chosen for young children with DSD, in part, because this is when some surgeons recommend such procedures [4] but also because caregivers believe that early surgery will spare their child from future problems. In other words, caregivers may see surgery as a way to protect their child and to exert control over their child's long-term medical prognosis and psychosocial adjustment. This speculation is testable and should be studied further.

The results regarding parenting stress also partially supported our hypothesis. Caregivers of adolescents experienced significantly higher rates of parenting stress than those of school-age children, but caregivers of infants and toddlers did not experience more stress than the other groups. Upon examination of the parenting stress subscales, caregivers of adolescents were found to experience higher rates of personal distress and distress related to their interaction with their child than the other groups, but no differences were observed between the groups on the difficult child subscale. These results suggest that much of the stress caregivers of adolescents with DSD are experiencing is not due to their child exhibiting particularly challenging behaviors, but it is due to their own personal distress (e.g., *I feel trapped by my responsibilities as parent*) and challenging interactions with their child (e.g., *Most times I feel that my child does not like me and does not want to be close to me*).

These results argue against the notion that caregivers are reacting negatively to their child's gender or sexuality. Rather, their stress may originate from personal maladjustment (e.g., depressive symptoms) and perhaps their own perceived difficulties in parenting their child. Again, we can speculate about DSD-specific scenarios that may increase caregiver distress in this way. For example, caregivers of older children may be faced with educating their child about their medical history, fertility, and long-term prognosis, and perhaps, they feel unprepared to do so. Challenging caregiver-child interactions may also be a result of negative feelings experienced by caregivers and fear of disclosing information to adolescents regarding their illness. There may be tension in their interactions because the adolescents sense that their caregivers are withholding information. By comparing parenting stress levels among caregivers who have educated their child prior to adolescence with caregivers who have not done so, this possibility could be tested. Another important study would be to compare caregivers who take advantage of DSD support groups to those who do not in order to determine if such activities increase their confidence in speaking candidly with and caring for an adolescent with a DSD.

Certainly, the current study has a number of limitations, including its cross-sectional design, exclusive use of self-report methodology, and relatively small sample size. Further, the study examined caregivers of children with several different medical conditions which fall under the umbrella of DSD. In addition, the lack of a control group precludes any conclusions that can be drawn concerning the uniqueness of this finding to caregivers of children with a DSD, though the extant literature would suggest that the finding regarding parenting stress in adolescents is unique [12]. Notwithstanding these limitations, this study is the first to address possible differences in caregiver adjustment to DSD as a function of developmental level of the child.

Overall, the current results suggest that caregivers of children with DSD experience different parenting challenges across their child's development. Understanding the unique challenges faced by caregivers of children with DSD relative to their child's developmental stage in the context of gender development will help to inform the development of optimal resources and support services for these caregivers and their families. In regard to clinical implications, when children are very young, caregivers may benefit from education regarding their child's diagnosis and developmentally appropriate expectations of their child. They may also benefit from support groups and mental health services targeted at normalizing their experience to decrease their need to shelter and protect their children. When children reach adolescence, caregivers may benefit from interventions targeted at their own distress as a parent, but perhaps more importantly, interventions designed to enhance communication between adolescents with DSD and their caregivers are warranted. This will help to resolve dysfunctional interactions between caregivers and adolescents and facilitate open communication regarding the child's diagnosis, fertility, and long-term prognosis.

## Competing interests

The authors declare that they have no competing interests.

## Authors' contributions

SH conceived of the study, performed statistical analyses, and drafted the manuscript. DF performed statistical analyses and drafted the manuscript. CW collected the data and revised the manuscript. LM participated in the design and coordination of the study and revised the manuscript. AW participated in the design and coordination of the study and drafted the manuscript. All authors read and approved the final manuscript.
